# Enabling interdisciplinary research capacity for sustainable development: self-evaluation of the Blue Communities project in the UK and Southeast Asia

**DOI:** 10.14324/111.444/ucloe.1970

**Published:** 2024-07-04

**Authors:** Fiona Culhane, Victoria Cheung, Melanie Austen

**Affiliations:** 1 School of Biological and Marine Science, University of Plymouth, Plymouth, UK

**Keywords:** interdisciplinary, transdisciplinary, marine and coastal ecosystems, research culture, environmental sustainability

## Abstract

Global challenges such as climate change, food security and human health and well-being disproportionately impact people from low-income countries. These challenges are complex and require an international and transdisciplinary approach to research, with research skills and expertise from different disciplines, sectors and regions. In addressing this, a key goal of the research project, Blue Communities, was to create and expand mutual interdisciplinary capacity of both United Kingdom and Southeast Asian Partners. An existing questionnaire on research capacity was uniquely adapted to include interdisciplinary and international aspects and distributed for the first time as an online survey to the participants of the Blue Communities project comprising researchers across all career stages. Participants were asked about their perceptions of the research capacity and culture of their organisation, team and self and whether they believed any aspects have changed since their involvement with the project. Greatest improvement was seen at the self-level where results indicated a positive relationship between an individual’s current success or skill and their improvement over the course of the research project across 18 out of 22 aspects of research capacity for Southeast Asian, and two for UK respondents. The conflict between achieving research aims, building research capacity and making societal impact was evident. Institutional support is required to value these core aspects of interdisciplinary research.

## Introduction

Global challenges such as climate change, food security and human health and well-being disproportionately impact people from low-income countries (LICs) [1] and are addressed through global governance with the United Nations Sustainable Development Goals (UN SDGs) [2,3]. It is increasingly recognised in the research community, by research funders (e.g., the UK’s Global Challenges Research Fund)^1^ and by institutions (e.g., universities) that these challenges are complex and require an international and interdisciplinary approach to research, integrating research skills and expertise from different disciplines, sectors and regions [4,5]. Building from a zero or near zero situation and/or strengthening existing sustainable capacity in research communities is required to address these global challenges [4], and we use these terms interchangeably hereafter. With finance and research agendas dominated by the Global North [6,7], research capacity is recognised to be unevenly distributed and often limited in the regions where global challenges are most felt [8]. Research programmes aimed at addressing global challenges therefore increasingly try to embed research capacity building and/or strengthening [8]. Capacity building must increase the resilience of the individual and/or organisation, thereby ensuring their longer-term sustainability [9] to address complex global challenges.

The often-uneven coverage of global challenges research between high-income countries (HICs) and LICs is exemplified by ecosystem service research, a key link between ecosystems and human well-being, which is lacking in Southeast (SE) Asian countries [10]. Collaboration between HICs and LICs has been suggested as a way to increase research capacity across all partners and to fill such research gaps [11,12]. However, studies have shown that research capacity building in such collaborations can be limited, for example, publications are often led by authors in HIC [5,8]. Nevertheless, it should also be noted that outputs of research publications and research funding, driven largely by the funders and the research culture in HICs, are not the only indication of research capacity [13,14]. Achieving these research products, can be in conflict with building research capacity [6,8]. In addition, the UK perception of ‘good’ research may contrast with perceptions of those in other cultures [15]. Harvey et al. [8] argue that significant disruption of the current system is required to truly achieve balanced research capacity.

The Blue Communities (BC) interdisciplinary research and capacity building project recognised that marine and coastal ecosystems are essential for food security, livelihoods, health and well-being through direct human activities such as fisheries and tourism, and for regulating and supporting services such as climate regulation; and that global loss of biodiversity and ecosystem services should be addressed through an integrated approach [16].^2^ BC was a four-year project, funded by the UK’s Global Challenges Research Fund (GCRF), that aimed to build capacity for sustainable interactions with marine ecosystems for health, well-being, food security and livelihoods. The primary objectives were to:

1. develop collaborative interdisciplinary research to improve the integrated management of marine and coastal environments to reduce conflict between users, mitigate risks associated with expanded or new uses, and protect fragile ecosystems while supporting livelihoods, food security, health and well-being of coastal communities.
2. create and expand mutual interdisciplinary capacity and capability building of both UK and SE Asian Partners and the study communities in integrated planning through sustainable interactions with marine ecosystems for the health, well-being, food and livelihoods of coastal communities.

The GCRF sought to achieve ‘meaningful and equitable relationships’ [17] through the goal of building research capacity across partners involved in the projects they funded. In the BC project, ‘a ‘learn by doing’ approach, where SE Asian researchers were encouraged to lead their research studies and seek support from experienced UK researchers when needed’ was taken (Blue Communities Handbook). Throughout the project, BC activities (e.g., skills workshops, paper writing, seminars, mentorship, flexible communication, networking, formation of research ethics and health and safety committees, etc.) allowed the building of research capacity, while achieving research objectives. The project also formed an Early Career Researcher network and encouraged Early Career Researchers to develop their own funding calls, proposals and apply for additional funding that had been set aside from the original core budget to support these.

The success of this approach can be evaluated by looking at the research products; however, this will only capture the current research outputs and not the sustainable future research capacity that has been built through the project. By taking a broader perspective on research capacity from a diverse group of researchers and allowing researchers involved in the project to have an opportunity to formally reflect on and report their perceptions of how research capacity has improved through involvement with the project, we are able to gain a fuller understanding of research capacity within the group. This learning can be used to enhance or modify approaches used for capacity building in future collaborations.

The aims of this paper are to:

- evaluate the perceptions of the current research capacity of the organisations, research teams and individuals involved in the BC project and identify potential strengths and gaps;
- evaluate the perceptions of the change in the research capacity of the organisations, research teams and individuals attributed to involvement in BC, and link this to the approach used by the BC research programme;
- explore demographic factors, particularly region, that may influence these perceptions;
- evaluate the successes and challenges and their implications for growing current and future research capacity for sustainable development.

## Methods

### Questionnaire

The questionnaire was based on the Research Capacity and Culture Tool [18], which gathers information on participant’s perceptions of the research capacity and culture of their institution, team and self across a range of generic research capacity markers. This questionnaire was adapted by the authors to be relevant to the researchers in this project. Specifically, additional markers for assessment were added, including on interdisciplinary and international working, carrying out research that has an impact and a question about the effect of the coronavirus (Covid-19) pandemic. Further open and closed questions were added to gain more in-depth insight into the perspectives of the project participants and how these aligned with the overarching aims of the project and the work that was carried out during the project. The questionnaire was held on the JISC online platform, and the link distributed by email to the members of the BC project. Project members were mainly from academic institutions and non-governmental organisations in the UK and in four SE Asian countries – Malaysia, the Philippines, Indonesia and Vietnam. Researchers within the project ranged from those with little research experience to those with long careers in research, and categories in the survey were chosen to capture all of these career stages. The survey was distributed in February 2022 and was open for two weeks. The timing of the distribution of the survey coincided with the final two months of the four-year BC grant and therefore captured perceptions at this point in time. The survey was written in the English language and consisted of questions in four parts: (1) demography, (2) individual research capacity, (3) team level research capacity (participant’s BC team at their own institution) and (4) institution level research capacity. Questions included those with a numeric scale response to rate skills on various aspects related to research capacity and rating scale responses to assess change in research capacity. See Appendix for the full survey.

### Data analysis

The demographic factor of main interest was the broad region of the respondent. To explore overall perceptions of research capacity and whether these differed between groups based on region (Global South and Global North), quantitative data were summarised based on the country of participant, or UK (/European) vs. SE Asian. Other demographic variables (gender, age, career stage/research experience and contract type) were also explored for associations with different responses to perceptions of research capacity. Due to small cell sizes, Fisher’s exact test was used to explore associations between variables throughout, with *p* values reported and significance taken at the 0.05 level.

To compare across unequal groups of responses to questions on what activities people participated in, what resources they benefited from, what are their motivators and barriers to carrying out research, and what they valued most from the project, responses were weighted according to the total number of individuals per group. That is, the frequency of responses is shown as the proportion of participants in a group who responded. These are presented as bar plots. Where response rates were low in certain groups, categories were combined as indicated (e.g., undergraduate plus MSc research experience).

The responses to a number of statements regarding participants’ experience in the project is visualised in side-by-side matrix plots where the size and colour of squares represent the frequency of responses against each score to each aspect of research capacity for UK (and other European) and SE Asian respondents. Matrix plots were produced using Raw Graphs 2.0.^3^

The relationship between the current research capacity (current success or skill across a range of aspects) and perceived improvement in capacity of these, was explored through Spearman rank correlation for the UK (and other) and the SE Asian regions. Correlation plots, *R* and *p* values indicate the strength of association between the current perceived research capacity and the perceived improvement of each aspect as a result of involvement in the Blue Communities project. A significant positive association (significant *p* value and positive *R* value) may indicate that higher levels of research capacity have resulted from involvement with the project. These were produced using ggplot2 (Wickham [19]) in *R* (R Core Team [20]). Significance was taken at the 0.05 level.

## Results

### Demographic information

A total of 56 people responded to the survey, out of approximately 115 researchers who were involved over various time periods throughout the project. Of these, most (57%) were female and aged between 31 and 50 years of age (64%) (Table 1). The largest group of respondents came from the UK (or other European countries) and the smallest from Indonesia.

**Table 1.** Demographics of the BC research community who responded to the online survey with information on the total population of the BC project, where available, for comparison

Demographic variable	Category	Proportion of respondents (%)	Number of individual respondents	Total number of individuals in BC project (proportion)
Gender	Female	57	32	59 (51%)
	Male	41	23	56 (49%)
	Prefer not to say	2	1	
Age range*	18–30	16	9	-
	31–50	64	36	-
	51+	18	10	-
	Prefer not to say	2	1	-
Country of Institution	Indonesia	7	4	16 (14%)
	Malaysia	20	11	19 (17%)
	Philippines	23	13	22 (19%)
	UK (and other European)	33	18	42 (37%)
	Vietnam	18	10	16 (14%)

*Four age categories were recorded in the survey, but due to low response 51–64 and 65+ categories were merged.

Most respondents to the survey came from academia (88%), though non-governmental organisations (NGOs) and government agencies were also represented (Table 2). Fifty-five per cent of researchers had fixed-term contracts and 74% had multiple work commitments. All career stages from early, mid and later career were represented in the survey, with 53% from the broader early career categories (students and PhD + five years or less experience).

**Table 2.** Information about the career type, stage and formal research experience of the BC research community who responded to the online survey

Variable	Category	Response rate (%)	Number of individuals
Sector	Academia	88	49
	NGO	9	5
	Other (Government Agency)	4	2
Contract type	Fixed term	55	31
	Permanent	45	25
Research experience*	Undergraduate degree and/or current MSc student	14	8
	MSc and/or current PhD student	25	14
	PhD with up to 5 years	14	8
	More than 5–15 years post PhD	29	16
	More than 15 years post PhD	18	10
Type of involvement in BC project	I work only on the Blue Communities project or Blue Communities is my main research project.	27	15
	My time is divided amongst multiple research projects, of which Blue Communities is one.	23	13
	Blue Communities is my only research project, but I also have other work commitments such as teaching or administrative work.	9	5
	My time is divided amongst multiple research projects, of which Blue Communities is one, and I also have other work commitments such as teaching or administrative work.	42	23

*Research experience had seven separate categories in the original survey, but due to low response rate in some groups undergraduate degree was merged with current MSc student; and MSc was merged with current PhD student.

There was evidence of an association between age and gender (*p* = 0.01), with more younger researchers being female; and age and experience (*p* < 0.01), with older researchers having more experience (for full results see Table A1). There was also an association between experience and country (*p* = 0.01) or region (i.e., UK and other vs. SE Asia; *p* = 0.02), with researchers with less experience being more likely to be from SE Asian countries.

### Individual research capacity

**Figure 1:**
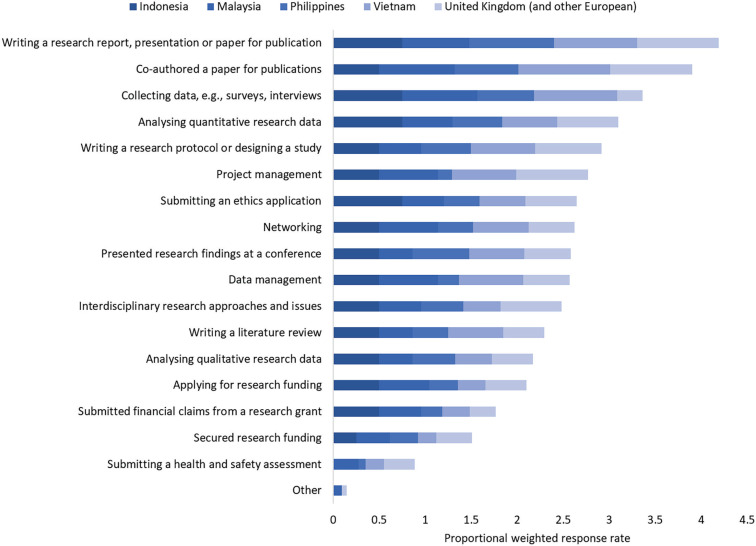
Research activities respondents have been involved with as part of the BC project. Respondents could choose as many options as were relevant. The bars are weighted according to the total number of respondents from each country/region (e.g., if every respondent chose an option, each bar segment would have a value of 1).

Respondents took part in a broad range of activities throughout the project, with most people involved in publishing, presenting, analysing quantitative data, collecting data and designing studies (Fig. 1). There was no evidence of an association with the type of activities carried out and gender (*p* = 0.987), age (*p* = 0.984), experience (*p* = 1), contract type (*p* = 0.998) and country (*p* = 1) or region (*p* = 0.811) (see also Table A2). Most researchers were involved in particular with writing reports (86%) and publications (82%), collecting (61%) and analysing (61%) data and designing studies (61%). Fewer people overall were involved with applying for and securing research funding (41%), submitting financial claims (32%) and submitting health and safety assessments (21%).

**Figure 2:**
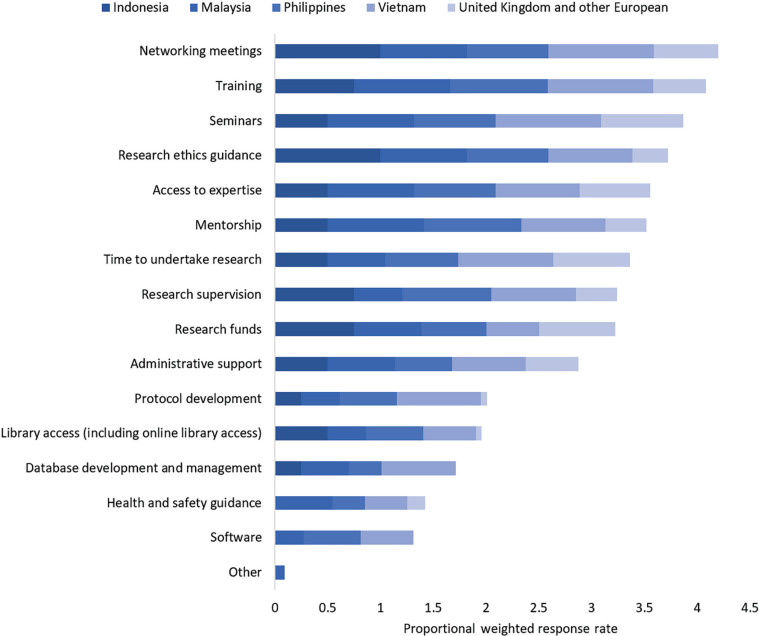
Resources respondents benefited from through the BC partnership. Respondents could choose as many options as were relevant. The bars are weighted according to the total number of respondents from each country/region (e.g., if every respondent chose an option, each bar segment would have a value of 1).

The resources researchers benefited from were associated with the region (*p* = 0.002, Table A2). Respondents across all regions benefitted the most from knowledge exchange resources such as seminars (80%), networking (79%), training (79%), access to expertise (73%) and mentorship (70%) (Fig. 2). Resources such as protocol development (38%), library access (34%), health and safety guidance (30%), database management (30%) and software (27%) benefitted fewer respondents overall, but of those, benefits were felt mostly by the SE Asian respondents.

**Figure 3:**
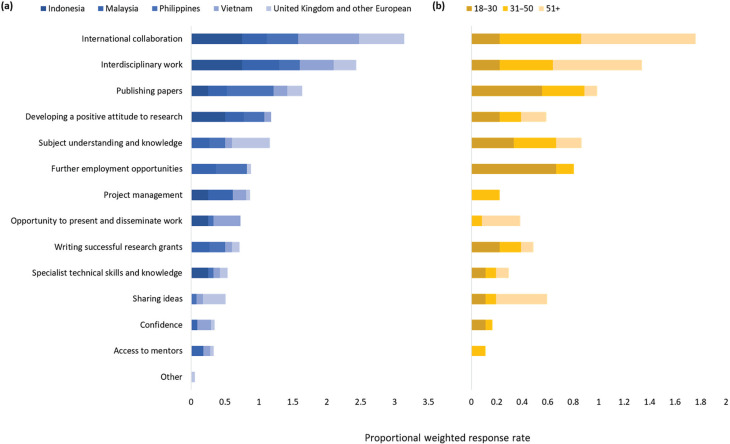
Research skills or opportunities respondents valued the most from their experience in BC. Respondents could choose up to three options. The bars are weighted according to the total number of respondents from (a) each country/region, and (b) their age (e.g., if every respondent chose an option, each bar segment would have a value of 1.

When asked what the respondents valued most from their BC experience, respondents across all across regions and career stages most valued interdisciplinary (61%) and international working (43%), publishing papers (34%) and improving their subject understanding and knowledge (30%) (Fig. 3). There was evidence of an association between age and the skills and opportunities valued (*p* = 0.023, Table A2), younger researchers in particular valued publishing papers and further employment opportunities. Country (*p* = 0.030) and region (*p* = 0.005) also had an association with values, with SE Asian researchers being more associated with valuing developing a positive attitude to research.

**Figure 4:**
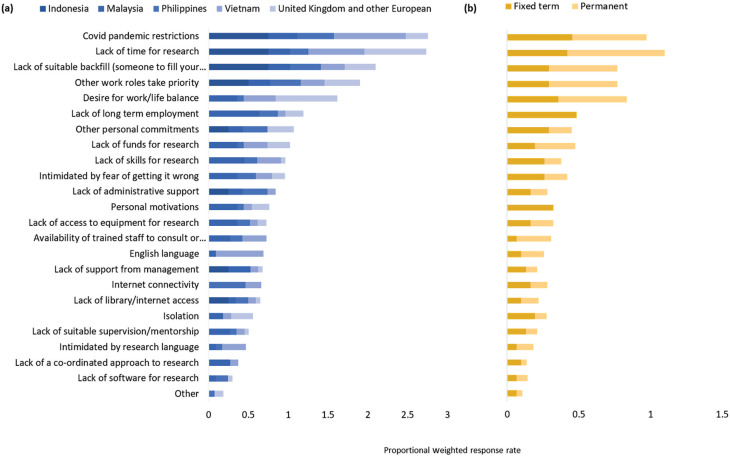
Barriers to research, according to participants of the BC project. Respondents could choose as many options as were relevant. The bars are weighted according to the total number of respondents from (a) each country/region, and (b) their contract type (e.g., if every respondent chose an option, each bar segment would have a value of 1).

Many of the top barriers to research that respondents identified were related to time constraints in general [e.g., ‘Lack of time for research’ (54%), ‘Desire for work/life balance’ (41%), ‘Other work roles take priority’ (38%) and ‘Lack of suitable backfill’ (38%)] (Fig. 4). There was an association with the contract type (*p* = 0.009, Table A2), with those on fixed-term contracts particularly identifying lack of long-term employment and personal motivations as barriers. Covid-19 pandemic restrictions were also identified as a key barrier by 48% of respondents, particularly for SE Asian researchers (*p* = 0.001). Other barriers were a lack of long-term employment (27%), personal commitments (23%), fear of getting it wrong (21%) and lack of skills (20%). English language was identified by 13% of respondents as being a barrier.

**Figure 5:**
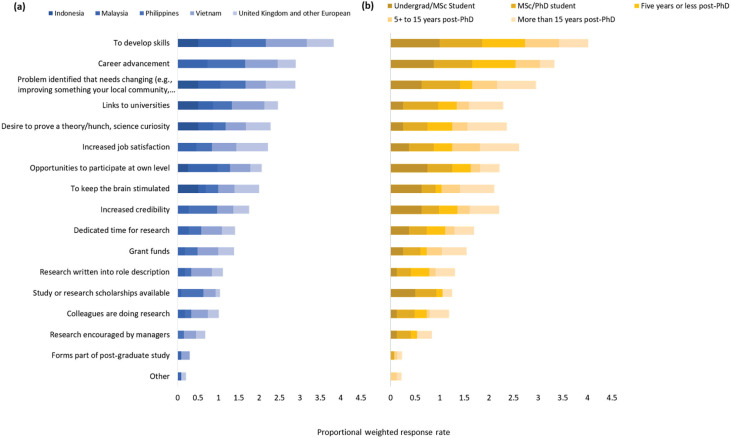
Personal motivators to research, according to participants of the BC project. Respondents could choose as many options as were relevant. The bars are weighted according to the total number of respondents from (a) each country/region, and (b) their career stage (e.g., if every respondent chose an option, each bar segment would have a value of 1).

When asked what personally motivates them to carry out research, respondents indicated developing skills (79%), advancing their career (64%), making an impact (a problem that needs solving) (61%), increased job satisfaction (54%) and scientific curiosity (46%) (Fig. 5). These options were indicated across gender, age, contract type, regional and career stage groups showing the motivations for research were common across this group of researchers (Table A2).

**Figure 6:**
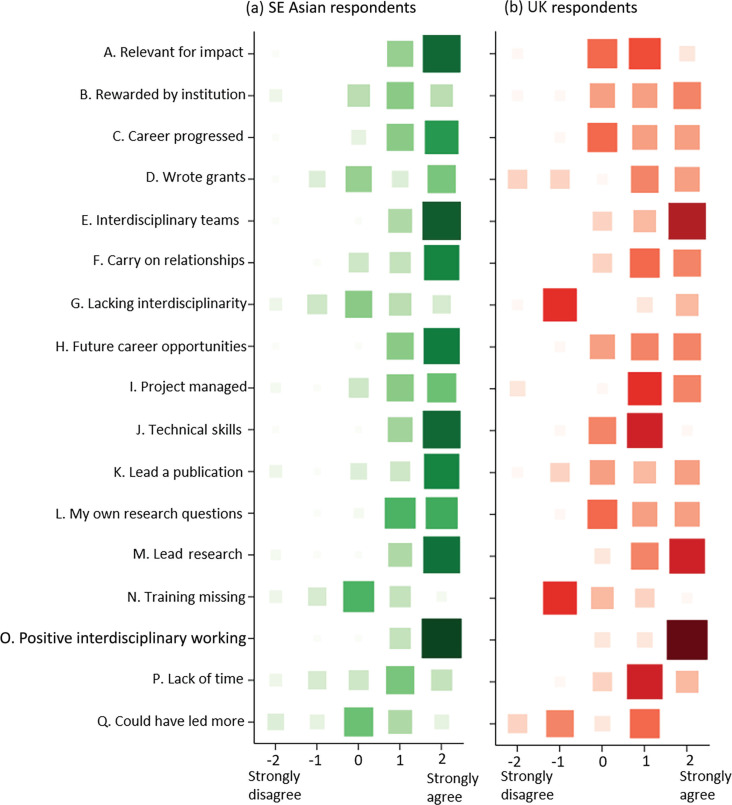
Level of agreement to a number of statements from (a) SE Asian, and (b) UK (and other European) respondents. A five-point scale was used: Strongly disagree (−2), Disagree (−1), Neither agree nor disagree (0), Agree (1) and Strongly agree (2). Larger square and darker colour indicates higher frequency of responses in the matrix plot. Statements A–Q are abbreviated in the figure, full statements and percentage breakdowns are given in Table A4 in the Appendix.

Across both broad regions, 91% of respondents agreed that they worked with interdisciplinary teams, with 66% in strong agreement with this statement (Fig. 6E); 91% agreed or strongly agreed that they feel positive about working with people from different disciplines in the future (Fig. 6O) and 89% that they had the opportunity to lead research (Fig. 6M). Sixty-eight per cent of respondents agreed or strongly agreed that they had the chance to lead a publication (Fig. 6K), of these 76% were from SE Asia. Leading publications was associated with age (*p* = 0.012; Table A3) and career stage (*p* = 0.021), with the youngest and least experienced, and oldest and most experienced not having led publications. On the whole, respondents from SE Asia responded more positively across all statements. Ninety-seven per cent of respondents from SE Asia agreed or strongly agreed that their research was relevant for making an impact in their region (making a difference to society), but this was less clear for UK respondents with 56% in agreement with this statement (Fig. 6A; *p* < 0.001 Tables A3 and A4). Ninety-two per cent of SE Asian respondents also agreed that they led on their own research questions (Fig. 6L; *p* = 0.008), compared to 56% of UK respondents. Ninety-five per cent also agreed they learnt new skills (Fig. 6J, *p* < 0.001), compared to 61% for UK respondents. SE Asian respondents also perceived that their career progressed, and prospects improved [Fig. 6C (88%), Fig. 6H (95%); *p* = 0.041, *p* = 0.015]. Fifty-six per cent and 67% of UK respondents agreed to the same markers on career progression and prospects.

**Figure 7:**
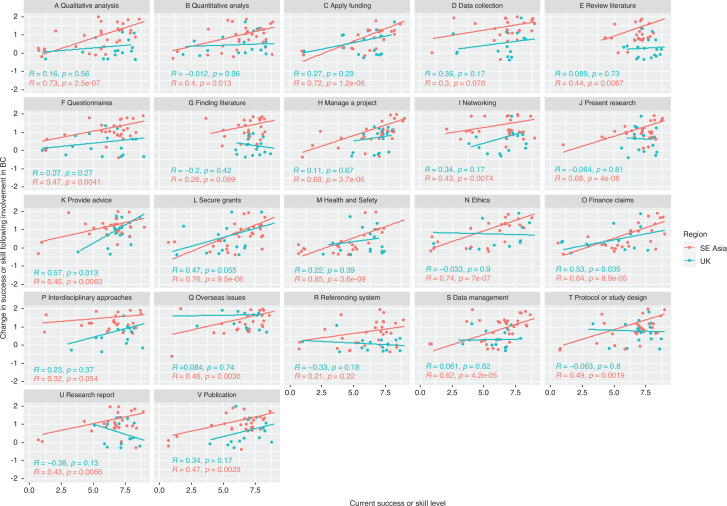
The relationship between SE Asian respondent and UK (and other European) respondent perceptions of their personal (individual level) current success or skill level for each aspect of research capacity (1 = no success/skill and 9 = highest possible success/skill) and change in success or skill level for each aspect as a result of involvement in the BC project (rating scale categories converted to numbers where –2 is ‘Much worse’, 0 is ‘no change’ and +2 is ‘Much better’). Correlation line, *R* and *p* values indicate Spearman’s rank correlation. Note that discrete data points are ‘jittered’ for visualisation purposes. Research capacity aspects A–V are abbreviated in the figure, full statements given in Table A6 in the Appendix.

At the individual level, across both broad regions, most respondents were confident in their success and/or skill on most aspects of research capacity, with 64% of ratings across skills being at a score of 7 or higher (Fig. 7), and with no sufficient evidence of a difference in success or skill between the regions on any aspect (Table A5). Respondents in both regions were most confident in finding and critically reviewing literature (Fig. 7E, G) with 84% scoring themselves 7 or higher. Seventy-nine per cent of respondents scored 7 or higher in presenting research (Fig. 7J) and 77% in protocol/study design (Fig. 7T). Sevent-five per cent scored 7 or higher in understanding interdisciplinary approaches and issues (Fig. 7P). Areas of lower confidence for respondents were in submitting a health and safety assessment (Fig. 7M; 32% scored 7+), financial claims (Fig. 7O; 41% scored 7+), in securing research funding (Fig. 7L; 45% scored 7+) and in submitting ethics applications (Fig. 7N; 52% scored 7+).

Self-assessed success or skill in the different aspects generally was not associated with demographic variables, except in a few circumstances. There was evidence of association with age and data collection (*p* = 0.05, Table A5), where the 31–50-year-old age category scored themselves highest; and age and reviewing literature (*p* = 0.04), where older age categories scored themselves higher. Early career researchers (up to PhD student) scored themselves lower on finding literature (*p* = 0.02) and on publishing (*p* = 0.04). There was an association with gender and the scores on quantitative analysis, where some female researchers scored themselves very low (*p* < 0.001).

In terms of change following involvement with the BC project, all but one respondent saw improvement in the understanding of overseas issues (Fig. 7Q). SE Asian partners indicated higher improvement across 14 out of 22 markers of research capacity compared to UK partners who mainly indicated no change or a smaller degree of improvement across most markers (Fig. 7, Table A5). SE Asian respondents saw greater improvement in collecting data (Fig. 7D, *p* < 0.001), finding and critically reviewing literature (Fig. 7G, *p* < 0.001, Fig. 7E, *p* < 0.001), questionnaires (Fig. 7F, *p* < 0.001), managing projects (Fig. 7H, *p* = 0.018), presenting research (Fig. 7J, *p* = 0.008), networking (Fig. 7I, *p* < 0.001), referencing and data management systems (Fig. 7R, *p* = 0.001, Fig. 7S, *p* = 0.027), research reports and publications (Fig. 7U, *p* = 0.002, Fig. 7V, *p* = 0.008) and understanding interdisciplinary approaches and issues (Fig. 7P, *p* = 0.001). Similar to UK respondents, they mostly saw no change submitting health and safety applications (Fig. 7M, *p* = 0.51) and in financial claims (Fig. 7O, *p* = 0.12). There was no association between other demographic variables and the degree of improvement reported.

There was evidence to suggest a significant positive correlation between the current success or skill of individuals and the degree of improvement during the BC project in 18 out 22 aspects for SE Asian respondents and in two aspects [providing advice (Fig. 7K) and submitting finance claims (Fig. 7O)] for UK participants (Fig. 7). Together this evidence indicates that SE Asian respondents, on most aspects, perceived that they had improved from a lower success or skill level to achieve a success or skill level that was in line with what UK respondents had assigned themselves from the start of the project.

### Team level research capacity

**Figure 8:**
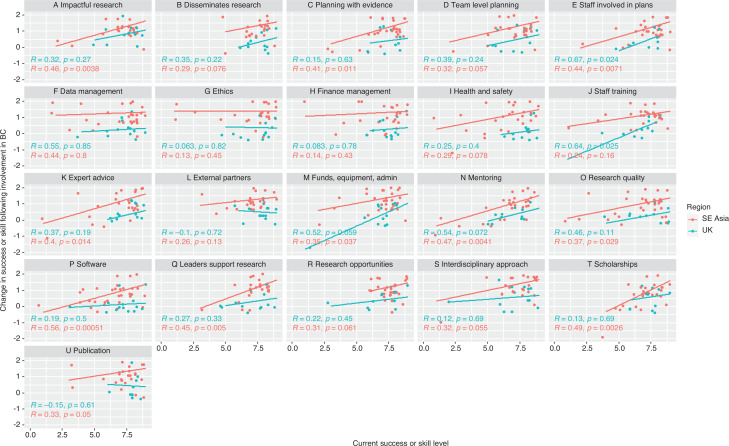
The relationship between SE Asian respondent and UK (and other European) respondent perceptions of their team’s current success or skill level for each aspect of research capacity (1 = no success/skill and 9 = highest possible success/skill) and change in success or skill level for each aspect as a result of involvement in the BC project (rating scale categories converted to numbers where –2 is ‘Much worse’, 0 is ‘no change’ and +2 is ‘Much better’). Correlation line, *R* and *p* values indicate Spearman’s rank correlation. Note that discrete data points are ‘jittered’ for visualisation purposes. Research capacity aspects A–U are abbreviated in the figure, full statements given in Table A8 in the Appendix.

At the team level (the participant’s BC team at their own institution), most respondents across both broad regions were confident in the success or skill of their team across most research capacity markers, with 74% of ratings across skills being at a score of 7 or higher and with insufficient evidence of a difference in success or skill between the regions on any aspect (Fig. 8, Table A7). Eighty-six per cent of respondents scored their team 7 or higher for publications (Fig. 8U), 82% for research opportunities (Fig. 8R) and 80% for having leaders that support research (Fig. 8Q). On other aspects, there was lower confidence with 63% scoring their team 7 or higher for having incentives and support for mentoring (Fig. 8N) and for availability of software to support research activities (Fig. 8P), and 64% for having adequate resources to support staff training (Fig. 8J). There was evidence of an association with career stage and disseminating research (Fig. 8B, *p* = 0.044), with early career groups (up to 5 years post PhD) scoring their teams highly on this; their team’s success in providing expert advice (Fig. 8K, *p* = 0.010), with MSc/PhD students scoring their teams lower on this, and scholarships (Fig. 8T, *p* = 0.041), with MSc/PhD students and those up to 5 years post PhD scoring their teams lower on this. More experienced researchers (*p* = 0.007) and those on permanent contracts (*p* = 0.035) scored their teams higher on software (Fig. 8P). Male researchers were associated with a lower team score for engaging with external partners (Fig. 8L, *p* = 0.025).

In terms of change following involvement with BC, there was disparity between groups, with SE Asian partners finding most aspects to be better or much better and UK respondents mostly reporting no change (Fig. 8). SE Asian respondents reported significantly higher improvement than UK respondents on all aspects except scholarships (T) (Table A7). There was no association with age, gender, career stage or contract type and the level of improvement.

There was evidence to suggest a significant positive correlation between the current success or skill of teams and the degree of improvement during the BC project in 11 out 21 aspects for SE Asian respondents and in two aspects [staff being involved in research planning (Fig. 8D) and staff training (Fig. 8J)] for UK respondents (Fig. 8). Together this evidence indicates that SE Asian respondents, on around half of research capacity markers, perceived that their teams had improved from a lower success or skill level to achieve a success or skill level that was in line with what UK respondents had assigned their teams from the start of the project.

### Organisational level research capacity

**Figure 9:**
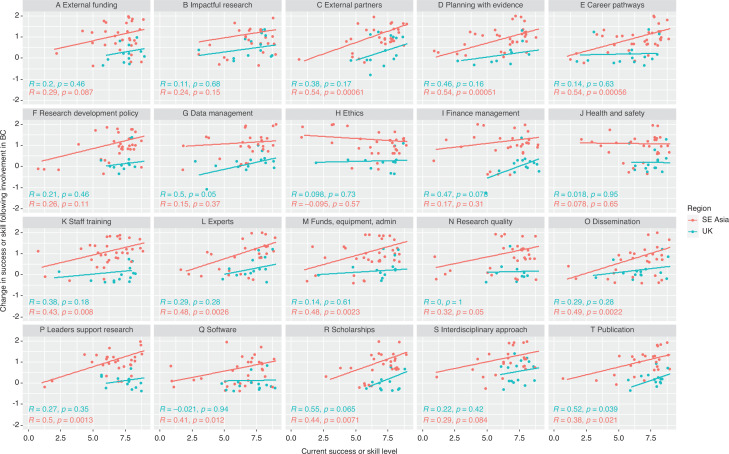
The relationship between SE Asian respondent and UK (and other European) respondent perceptions of their organisation’s current success or skill level for each aspect of research capacity (1 = no success/skill and 9 = highest possible success/skill) and change in success or skill level for each aspect as a result of involvement in the BC project (rating scale categories converted to numbers where –2 is ‘Much worse’, 0 is ‘no change’ and +2 is ‘Much better’). Correlation line, *R* and *p* values indicate Spearman’s rank correlation. Note that discrete data points are ‘jittered’ for visualisation purposes. Research capacity aspects A–T are abbreviated in the figure, full statements given in Table A10 in the Appendix.

At the organisational level, again most researchers rated their organisation’s success or skill highly across all or most research capacity markers in both broad regions, with 66% of ratings across skills being at a score of 7 or higher (Fig. 9). Seventy-seven per cent of respondents scored their institutions 7 or higher for accessing external funding for research (Fig. 9A), encouraging research activities relevant to creating impact (Fig. 9B), and for supporting the peer-reviewed publication of research (Fig. 9T). While only 54% of respondents scored their institutions 7 or higher for ensuring organisational planning is guided by evidence (Fig. 9D) and ensuring staff career pathways are available in research (Fig. 9E). Only for having adequate support for staff training (Fig. 9K), did UK respondents score their institutions higher than SE Asian respondents (*p* = 0.049, Table A9). For this aspect, 72% of UK respondents and 47% of SE Asian respondents scored their institutions 7 or higher. There was an association with career stage and scores attributed to some aspects. Later career researchers (more than 15 years post PhD), scored their institutions higher on getting external funding (Fig. 9A, *p* = 0.046), their institution’s access to software (Fig. 9Q, *p* = 0.011) and on its interdisciplinary approach (Fig. 9S, *p* = 0.041).

In terms of improvement following involvement with BC, SE Asian respondents reported some improvement (‘Better’) across all markers and overall higher improvement than UK respondents across all markers, who reported mostly no change (Fig. 9, Table A9). There was evidence of an association with gender on degree of improvement on two aspects, with females more likely to report no improvement at their institution for research development policy (Fig. 9F, *p* = 0.006) and ethics (Fig. 9H, *p* = 0.005).

There was evidence to suggest a significant positive correlation between the current success or skill of institutions and the degree of improvement during the BC project in 11 out 20 aspects for SE Asian respondents and in one aspect [publication (Fig. 9T)] for UK participants (Fig. 8). Together this evidence indicates that SE Asian respondents, on around half of the research capacity aspects, perceived that their institutions had improved from a lower success or skill level to achieve a success or skill level that was in line with what UK respondents had assigned their institutions from the start of the project.

## Discussion

This paper has presented quantitative data from a diverse group of researchers on the impact of the research capacity building activity in an internationally collaborative project that has taken the specific approach of ‘learning-by-doing’. Generally, this appears to have been a successful strategy based on the largely positive perceptions of the respondents to this survey but was particularly successful at the individual level with respondents from SE Asia, who attributed clear improvements across 18 out 22 aspects of research capacity to their involvement in the BC project. Here, evidence for building and strengthening of research capacity through this project was based on the perceptions of participants who were at the end of the four-year project period and is discussed in the important context of its sustainability into the future to address the ongoing global challenges.

### Successes, or what worked well for current and future research capacity

The skills and opportunities valued most by the respondents of this study were interdisciplinary (chosen by 43%) and international working (chosen by 61%) to make a difference to society and 91% felt positive about continuing to work in this way in the future; one respondent reflected on ‘working with amazing international partners on issues that matter’ (BC project participant, UK) and another could see an impact in their local community: ‘the great response of the communities to our engagements’ (BC project participant, Philippines). Almost all (97%) respondents from SE Asia could see that their research was relevant for making an impact in their region, while 61% of the full group identified a problem that needs to be solved as one of the motivations for their research. While some researchers recognised the challenges and benefits of this type of working, ‘Having differing disciplines within the team is enriching and engaging despite the conflicts that came with it’ (BC project participant, Malaysia), building trusting relationships between partners, with integration and collaboration, is one of the key requirements of a successful interdisciplinary capacity building project and keeping people engaged in the process [8,9,21,22]. Capacity building is not only about transferring traditional skills but also about ‘a process of strengthening relationships that enable innovation and resilience in communities, organisations and societies’ [9], thus, the process of collaborating and working together builds capacity in itself [17]. The results of this survey suggest that the researchers involved are enthusiastic, passionate and engaged in working collaboratively and making a difference to society. Importantly, respondents expressed their hopes for continuing to work this way in the future, with 77% hoping to build upon the networks and relationships that were developed through the project. As one respondent stated: ‘I hope to continue to cooperate in the future, to develop the research direction of the project’ (BC project participant, Vietnam).

One clear example of ‘learning-by-doing’ in action was in carrying out evidence synthesis and systematic reviews. During the project a team of UK researchers who are very experienced in systematic reviews ran a series of training sessions and provided ongoing guidance and support to SE Asian researchers in developing their own systematic reviews with research questions relevant for their region. This approach was clearly successful in that researchers in SE Asia identified critically reviewing literature as being a factor they are particularly skilled or successful at and identified this as an area of much improvement because of involvement with the project. Three systematic reviews were carried out for three of the SE Asian partner countries, all led by SE Asian researchers (publications in progress). In addition, protocols for carrying out reviews were also developed and published [23,24]. Furthermore, participants in the workshops have since gone on to teach the method to others in their institution, demonstrating the sustainable nature of this capacity building.

Notably, lead authorship in the BC project amongst the respondents was distributed between those from different countries, leaning more towards those from SE Asia, with 76% of SE Asian and 50% of UK respondents agreeing they had the opportunity to be a lead author. This was clearly appreciated by some, as one respondent described their team’s motivation as being ‘the independence granted to develop and pursue research questions’ (BC project participant, Indonesia). This is in contrast to many studies that show disparity in lead authorship between high- and low-income partner countries. For example, in the Future Climate for Africa programme, Harvey et al. [8] found only 14% of 230 publications were led by a researcher from an African institution. Interdisciplinary research, by nature, requires input from a diversity of partners coming from different knowledge backgrounds but power imbalances can mean that these different actors do not always contribute sufficiently [21]. A key feature of BC was that it was decided from the outset that early career researchers, in particular those from SE Asian partner institutions, would be prioritised in terms of leading research and publications, and were supported by more senior staff in doing this. In addition, the project established an Early Career Researcher Network, which encouraged members to apply for additional funding to support their own research questions, host seminars and share skills. Having this set out clearly and supported with leadership meant these power imbalances were explicitly addressed.

The Covid-19 pandemic restrictions presented a challenge, as reported by respondents, especially SE Asian participants (58% of SE Asian respondents identified this as a barrier to research). This was through an inability or reduced time to visit field sites and collect new data, an inability to meet project partners in person, and potentially more difficulty with Internet or resource access, as well as other personal factors. This is likely to have impacted capacity building through impacting development of personal relationships. Despite this, SE Asian partners responded positively in terms of improvement due to their involvement with the project across 18 out 22 research capacity markers. Teams adapted quickly to the new situation and in some cases changed their focus. Indeed, partners in the project demonstrated good practice in moving activities online in a sensitive and structured way [25]. In some, but not all cases, project participants recognised that they were fortunate to have the pandemic come later in the project so that personal relationships were already well established. However, where this was not the case, partners demonstrated concerted effort in building relationships online. For example, Richter et al. [25] emphasised the importance of using icebreakers in the virtual environment. This made a relatively smooth transition to moving capacity building elements and research working online.

Most respondents agreed or strongly agreed that they had the opportunity to lead research questions (80%) and publications (68%), they learnt new skills (84%), that their career level progressed (77%) and that they would have more career opportunities available (86%) to them as a result of their involvement in BC. This shows that the respondents perceive concrete and sustainable capacity building has been achieved during the project, and that partners feel they can carry on with this type of research independently into the future. One respondent reflected: ‘my involvement at the Blue Communities has increased my visibility in the local academia. This program has also significantly impacted my research and project management skills. Most importantly, my involvement with the Blue Communities has paved my career path in significant ways’ (BC project participant, Malaysia).

### Challenges for sustainable current and future research capacity

An issue identified previously in research projects that aim to create impact in solving global challenges and build capacity is the conflict between research aims (e.g., advancing knowledge and publishing papers), influencing policy and building capacity [8]. Harvey et al. acknowledge that a common strategy is often used to achieve these aims, but this may not be appropriate for all, and research aims can be given priority. This conflict clearly emerged during the BC project. Just over half of respondents to the survey were on fixed-term contracts and, traditionally, publishing papers is important for career advancement, while even established researchers depend on their publication record in winning further research funding. Younger researchers, in particular, valued publishing papers and further employment opportunities (56% and 67%, respectively, of 18–30 year olds valued these skills/opportunities), but publishing was important for many respondents, with several mentioning publishing papers as a motivator for their team, and one respondent describing the motivation to be the ‘Esteem and recognition for good research published, contributing to career development and attraction of further research funding for self-determined research pathways’ (BC project participant, UK). However, tension with these motivations and the aims of building capacity and achieving real impact in communities and how this is recognised for individuals, was also felt, as one respondent described: ‘I’d say some team members are too obsessed with papers as a marker of success, and universities do not sufficiently recognise the value of impact in their promotion criteria’ (BC project participant, UK).

This tension may be driven particularly by the UK side where researchers may feel under more pressure to publish for their career progression and to meet expectations of funding bodies. Fifty-six per cent of UK respondents agreed their career had progressed during the project compared to 87% of SE Asian respondents. One SE Asian respondent noted that ‘I’m now appointed as a Senior Lecturer at a local university, and one thing that got me into this job is because my employer values my networking with the international, multidisciplinary research team of BC’ (BC project participant, Malaysia) indicating that the values in UK universities differ from those that may be found in other cultures [15]. Overall, across almost all markers and at all levels, SE Asian participants reported more positive improvement than UK participants, who only identified improvements due to involvement with the project in, at most, two markers at individual, team or institutional level. Several factors may explain this, for example, the markers given may not capture adequately what UK participants may have benefited from nor what adequately evaluates interdisciplinary aspects of research capacity [21]. However, it could also be that in some cases participants felt capacity building was acting mainly in one direction. For example, only 56% of UK respondents agreed they had been able to answer some of their own research questions compared to 92% of SE Asian respondents. One respondent said ‘Compared to traditional research projects, the career progression opportunities for UK teams may have [conversely] advanced less. The focus was on capacity development, rightly, but this may have inadvertently reduced the scientific innovation and output from UK teams because of the amount of time needed to support the partner teams’ (BC project participant, UK). While UK respondents felt positively about some aspects, for example, 83% agreed that they project managed, if these attributes are not obviously valued in their career pathways, individuals may also not value these highly. Considering that interdisciplinary researchers tend to publish less at first and have greater difficulty in demonstrating research productivity than more traditional researchers [21], the perceived lack of career development in this type of project will only exacerbate the conflict between research aims, building capacity and making an impact. The increasing importance of impact in the UK’s evaluation of higher education providers through evaluations by funding bodies such as the UK Research and Innovation’s (UKRI) Research Excellence Framework and Knowledge Excellence Framework may go some way towards valuing and incentivising researchers who participate in capacity building research.

In some cases, within the project, researchers did prioritise research aims. Other studies of international consortia have reported that researchers in the Global South can feel like ‘data sources’ in that they are not heavily involved in planning or analysing data, but only in commenting on it; that responsibility stays in the North [8]. In the BC project, researchers from both regions were involved in the collection of data to some degree, and it was clear that SE Asian respondents were involved in all aspects of research, from planning, to collecting data, to analysing and interpreting. There were instances throughout the project where SE Asian partners sometimes deferred to UK partners to carry out complex analyses. For example, one respondent observed: ‘Some [sub-]projects, while providing training at annual meetings, ended up doing the analysis for the partners rather than training and then letting partners take ownership of the research. This is reflected in some [sub-]projects not having many papers lead authored by [SE Asian] partners’ (BC project participant, UK). Harvey et al. [8] emphasised the importance of being willing to fail as part of a learning-by-doing process, thus sometimes sacrificing high-impact research outputs to focus on capacity development.

It was unexpected that UK respondents did not feel more strongly that their research capacity improved due to their involvement with the project, in particular in relation to applying and understanding interdisciplinary approaches. A greater understanding of overseas issues was the only marker where all UK respondents identified improvement. This particular marker may encompass a multitude of factors, and it may be that the parameters provided in the survey do not adequately articulate what UK researchers did learn from involvement with the project. It is important to identify these parameters and ensure more active two-way dialogue in future collaborations, so that UK or other participants from HICs are mutually learning from their project partners. Although UK researchers may have seen themselves more in the role of delivering research capacity than receiving it, there are important reasons for mutual learning and capacity strengthening. Just over half of UK researchers identified the project as having an impact in their region. This is not totally unexpected since UK partners were not working directly with local communities as SE Asian partners were. However, there are areas that could have potential impact in the UK. For example, the current discourse in the UK on the need to decolonise the curriculum [26] would clearly benefit from researchers who have experience working with other cultures and introducing this diversity through their teaching and research citations. In addition, researchers working directly with communities in LICs on sustainability issues try to highlight the knowledge that is held in the Global South as ‘the limited Western view of sustainability is stifling progress’ [27]. SE Asian partners instigated a wealth of approaches throughout the project, working creatively with local communities and practitioners. For example, researchers in Indonesia carried out participatory film making with local communities addressing sustainability issues. This resulted in changes in environmental behaviours and the formation of a film making community group dedicated to making audio visual work on behavioural change related to plastic pollution and climate change. Another example from Malaysia saw engagement with local communities resulting in greater attendance to health centres and vaccine uptake. More work is needed to reflect on and recognise the learning of UK partners in this collaboration. However, this may become more apparent over the longer term than at the point this survey was carried out.

There was disparity in resources at organisational level between UK and SE Asia, with less than half of SE Asian respondents scoring their institutions highly for having adequate resources to support staff research training, while 72% of UK respondents reported their organisations were good in this. In other studies, participants have felt that it is important to recognise this organisational inequality to manage expectations and ensure a meaningful partnership [17]. The level of improvement at the institutional level was perceived by SE Asian respondents to be more limited than at the individual level, with improvement in only around half the markers correlating with the current success. Development is still needed at an institutional or organisational level to reduce inequality in these factors, as there can be a lack of investment at higher levels, beyond the individual [8]. Despite this, 79% of SE Asian and 72% of UK respondents felt that they would build upon the international networks and relationships developed through the project.

Many respondents felt lower confidence in submitting health and safety assessments, financial claims and ethics applications, though at an individual level, there were improvements in these for SE Asian respondents, and improvement in financial claims for UK respondents. At the team and institution level, these areas were not perceived to have improved. While not all respondents would have needed to participate in these aspects, and that may explain some of the variability, these aspects may reflect a lack of facilities or support for these within organisations but also that they can be complex administrative processes where rules can be unclear even where facilities are well developed. For example, one respondent mentioned the ‘bureaucracy of financial process’ (BC project participant, Philippines) as a barrier to their team. Additionally, ethics applications are often reviewed by individuals on an ethics committee and responses to applications can depend strongly on the individual reviewers, which can vary from organisation to organisation. Similar studies have also found efficiency of researchers to be inhibited by bureaucracy or technical and administrative support in time-limited research projects [8,17]. This project worked with organisations to develop their ethical approval processes, financial management and risk assessment, and there is variability in these depending on the specific location. One respondent mentioned a team barrier as being ‘lack of administrative support in the initial stage of project’ (BC project participant, Malaysia), indicating that things did improve. Despite lower confidence indicated by respondents on these aspects, from the personal observations of the principal investigator and project manager (authors MA and VC on this paper), there was substantial improvement of SE Asian individual, team and to some extent organisational capacity in financial claims and ethics processes. This project, through learning-by-doing, adapted a flexible approach, to meet the needs of researchers in different countries and organisations and adapt to their specific circumstances. This included, for example, providing advances on funding to allow participants to travel or take part in research activities and circumvent inhibitive administrative processes.

### Study limitations

There are limitations to this study, specifically that almost 90% of respondents came from academia, and to fully evaluate a transdisciplinary project, the perspectives of other actors, such as community partners, are also needed [21]. The objectives of other actors, or their perceived markers of success in research capacity needed to reach complex sustainability goals, are likely to differ from those with an academic focus, such as in terms of how capacity may translate to making an impact in communities, and this has not been captured in the responses to this survey.

The survey was only available in the English language, and this would have excluded some potential respondents. It is likely that the response to the English language acting as a barrier is an underestimate for this project, and ideally the survey would be translated to local languages to reach and get perspectives of all participants. For example, Indonesian respondents were underrepresented in the survey, and we are aware that some of the participants from Indonesia would have been restricted by the language barrier as they are non-English speakers. The BC project largely operated through English and non-English speakers relied on information being passed on by their colleagues. From this survey, we cannot say to what degree this knowledge transfer benefitted non-English speakers or if their research capacity improved. Future work should aim to assess this. Projects should ensure that local researchers form part of capacity building teams, and that ways to deliver knowledge and capacity in local languages are embedded within projects.

A longer-term assessment of research capacity will be required to evaluate if it has sustained into the future beyond the life of the project [14,28]. A key measure of research capacity is if it is lasting and if it can spread more widely in society. While this survey captured respondents’ perspectives at a specific time, just as the project was ending, this perspective could change over time, following experiences with transferring skills and knowledge to other projects or work.

### Lessons learnt and implications for future projects

This study provides a broader perspective on the success of a learning-by-doing approach to building research capacity than focussing on research outputs such as publications and funding alone. There are key lessons emerging from the outputs of this study that can be used to enhance or modify approaches used for capacity building in future collaborations:

- Identify the benefits that partners that are in the role of delivering research capacity training may receive from such partnerships, and the parameters to measure these benefits, to ensure that these are clearly recognised and therefore can be valued and incentivised in career paths.
- Explicitly address power imbalances. This can look like, at leadership level, deciding on a strategy that prioritises certain groups to be supported in leading research and publications, for example, researchers from LICs and early career researchers. This could also include taking a flexible approach and providing additional support for administration, for example, finance, ethics.
- Develop concrete tools/training that can be taught to and applied by participants within the time of the project, so that skills can then be passed on locally by those participants.
- From the outset, put effort into building relationships and establishing trust between partners. In the BC project, this was established through (i) sharing roles and responsibilities, for example, holding the kick-off meeting in SE Asia, co-organised by partners there, and early scheduling of presentations from all partners; (ii) establishing an inclusive project culture, for example, mixing of groups, listening, all questions valid, patience and understanding; (iii) finding common interests, for example, social interaction around food from different cultures; and (iv) maintaining communication, for example, with follow-up in-person and online meetings.

## Conclusions

There is currently a difficult balance between undertaking innovative interdisciplinary research that has societal impact and building sustainable research capacity. In this case, the BC project partners that responded to this survey perceived that the project achieved advances in all of these areas. This may provide lessons for other interdisciplinary research collaborations and capacity building efforts. The BC approach placed a strong emphasis on building relationships from the inception of and throughout the project, through a collaborative learning-by-doing process, that kept people enthusiastic and engaged to the end. However, gaps were identified by respondents in scientific innovation and in particular aspects of research capacity, and much of this may have arisen from trying to achieve what can be seen as conflicting aims. Despite the project recognising the importance of interactive dialogue and not just one-way training, for mutual capacity building [25], UK respondents reported less capacity built across most parameters. While this needs further investigation and other factors may come into play, this may in part be driven by the values of UK organisations. Institutions are responsible for incentivising individuals’ actions [9]. Currently, the incentives around research and career progression within research, particularly amongst HICs, are focused on publishing papers, and interdisciplinary researchers face challenges in having their achievements and skills recognised in traditional academic career paths [29–31]. Institutions and employers need to increase their efforts to place greater value on the contributions people make in the areas of strengthening capacity and making societal impact, giving it equal or higher value to research publications. This is essential to mobilising interdisciplinary and transdisciplinary research to solve global challenges and achieve long-term sustainability.

## Data Availability

The datasets generated during and/or analysed during the current study are available in the repository: https://www.doi.org/10.5255/UKDA-SN-856101.

## Notes

1 The Global Challenges Research Fund (GCRF) is a UK fund that promotes achievement of the UN SDGs in developing countries, through supporting international research. It is part of the UK’s Official Development Assistance (ODA) programme that aims to promote sustainable growth of the Organisation for Economic Cooperation and Development (OECD) selected developing countries. https://royalsociety.org/~/media/grants/schemes/ODA-GCRF.pdf?la=en-GB&hash=B51F1E2140346184856E2F87D6F4B32A.

2 https://www.plymouth.ac.uk/research/institutes/marine-institute/our-research/blue-communities.

3 https://rawgraphs.io/.

## Appendix

### Appendix A: Survey questions

**(note: numbers refer to the corresponding numbers in the open access data file)**

**Filter questions:**

**7. Do you currently or have you previously carried out research as part of the Blue Communities project?**

Yes/No

#### Section 1: Demographic questions

**8. What is your gender?** Male/Female/Prefer not to say

**9. What is your age group?** 18–30; 31–50; 51–64; 65+; Prefer not to say

**10. What sector do you work in?** Academia, NGO, other (please state if other)

**11. What research experience do you have?** Undergraduate degree; Current Masters student; Researcher (post Masters, no PhD); PhD student; </ = 5 years post PhD; >5–15 years post PhD; >15 years post PhD; other

**12. What is your contract type at your institution?** Fixed Term; Permanent

**13. In which country is your main institution located?** Indonesia; Malaysia; Philippines; United Kingdom; Vietnam

**14. Choose the option that best describes your association with the Blue Communities project** (for the majority of the time you have worked on the project):

- I work only on the Blue Communities project or Blue Communities is my main research project
- My time is divided amongst multiple research projects, of which Blue Communities is one
- Blue Communities is my only research project, but I also have other work commitments such as teaching or administrative work
- My time is divided amongst multiple research projects, of which Blue Communities is one and I also have other work commitments such as teaching or administrative work
- None of these options describe my association with the Blue Communities project

#### Section 2: Individual level

**15. Please indicate any research activity you are currently involved with or have been involved with as part of Blue Communities.** Tick as many as apply

- Writing a research report, presentation or paper for publication
- Writing a research protocol or designing a study
- Submitting an ethics application
- Submitting a health and safety assessment
- Collecting data, e.g., surveys, interviews
- Data management
- Analysing qualitative research data
- Analysing quantitative research data
- Writing a literature review
- Applying for research funding
- Networking
- Project management
- Interdisciplinary research approaches and issues
- Secured research funding
- Co-authored a paper for publications
- Presented research findings at a conference
- Submitted financial claims from a research grant
- Other

**16.**

- **Based on your perception, rate your personal current success or skill level for each of the following aspects** (1 = no success/skill and 9 = highest possible success/skill): 1–9/unsure
- **And secondly, say whether you think this aspect has changed as a result of involvement with the Blue Communities project** (on a scale of much worse – worse – no change – better – much better/unsure)
  16.1	Finding relevant literature
  16.2	Critically reviewing the literature
  16.3	Using a computer referencing system (e.g., Endnote)
  16.4	Writing a research protocol or designing a study
  16.5	Securing research funding
  16.6	Submitting an ethics application
  16.7	Submitting a health and safety assessment
  16.8	Submitting financial claims from a research grant
  16.9	Designing questionnaires
  16.10	Collecting data, e.g., surveys, interviews
  16.11	Using computer data management systems
  16.12	Analysing qualitative research data
  16.13	Analysing quantitative research data
  16.14	Writing a research report
  16.15	Writing for publication in peer-reviewed journals
  16.16	Providing advice to less experienced researchers
  16.17	Understanding interdisciplinary approaches and issues
  16.18	Understanding overseas issues and challenges
  16.19	Applying for research funding/writing research grants
  16.20	Networking
  16.21	Managing a project
  16.22	Presenting research findings

**17. Which of the following resources have you benefited from through the Blue Communities partnership?** Tick all that apply

- Software
- Research supervision
- Time to undertake research
- Research funds
- Administrative support
- Training
- Library access (including online library access)
- Protocol development
- Access to expertise
- Database development and management
- Health and safety guidance
- Research ethics guidance
- Seminars
- Networking meetings
- Mentorship
- Other (please state)

**18. What research skills or opportunities do you value the most from your experience in Blue Communities?** Tick up to three responses

Publishing papers; Writing successful research grants; Developing a positive attitude to research; Further employment opportunities; Subject understanding and knowledge; Confidence; Specialist technical skills and knowledge; International collaboration; Project management; Opportunity to present and disseminate work; Sharing ideas; Transdisciplinary work; Access to mentors; Other

**19. What are the barriers to research for you personally?** Tick all that apply

- Lack of time for research
- Lack of suitable backfill (someone to fill your other work commitments)
- Other work roles take priority
- Lack of funds for research
- Lack of support from management
- Lack of suitable supervision/mentorship
- Lack of access to equipment for research
- Lack of administrative support
- Lack of software for research
- Isolation
- Lack of library/internet access
- Personal motivations
- Other personal commitments
- Desire for work/life balance
- Lack of a co-ordinated approach to research
- Lack of skills for research
- Intimidated by research language
- Intimidated by fear of getting it wrong
- English language
- Covid-19 pandemic restrictions
- Availability of trained staff to consult or collaborate with
- Internet connectivity
- Lack of long-term employment
- Other (please state)

**20. What are your motivators to conduct research for you personally?** Tick all that apply

- To develop skills
- Career advancement
- Increased job satisfaction
- Study or research scholarships available
- Dedicated time for research
- Research written into role description
- Colleagues are doing research
- Research encouraged by managers
- Grant funds
- Links to universities
- Forms part of post graduate study
- Opportunities to participate at own level
- Problem identified that needs changing (e.g., improving something your local community, benefitting environment, etc.)
- Desire to prove a theory/hunch, science curiosity
- To keep the brain stimulated
- Increased credibility
- Other

**21. State how much you agree or disagree with the following statements as a result of your involvement in the Blue Communities programme** (Rating scale):

21.1	The research I carried out during Blue Communities was relevant to creating impact (e.g., making a difference to society, SDGs, local communities, policies, management, etc.) in my region
21.2	I had the opportunity to lead research work and/or contribute ideas that directed the research
21.3	I learned new technical specialist skills
21.4	I have had the opportunity to be the lead author on one/more than one publication
21.5	I project-managed
21.6	I did not have time to learn all that I might have during Blue Communities
21.7	I wrote new research grants during my time on Blue Communities
21.8	I worked with interdisciplinary teams
21.9	I felt some types of training were missing from the Blue Communities project
21.10	I feel positive about working with people from different disciplines in the future
21.11	I have been able to answer some of my own research questions
21.12	I will build upon the international networks and professional relationships that have been developed through the Blue Communities programme
21.13	I could have led more work than I did during the Blue Communities project
21.14	I think I will have more opportunities available to enhance my future career as a result of the work I have conducted for the Blue Communities programme
21.15	My career level has progressed as a result of my involvement in Blue Communities
21.16	I thought the Blue Communities research could have been more interdisciplinary
21.17	My institution rewards or recognises my achievements linked to Blue Communities

#### Section 3: Team level

**22.**

- **Based on your perception, rate your Blue Community team’s (at your own institute) current success or skill level for each of the following aspects** (1 = no success/skill and 9 = highest possible success/skill): 1–9/unsure
- **And secondly, say whether you think this aspect has improved as a result of involvement with the Blue Communities project** (on a scale of much worse – worse – no change – better – much better/unsure)
  22.1	Has adequate resources to support staff research training
  22.2	Has funds, equipment or admin to support research activities
  22.3	Does team level planning for research development
  22.4	Ensures staff involvement in developing that plan
  22.5	Has team leaders that support research
  22.6	Provides opportunities to get involved in research
  22.7	Does planning that is guided by evidence
  22.8	Conducts research activities relevant to creating impact (e.g., making a difference to society, SDGs, local communities, policies, management, etc.)
  22.9	Supports applications for research scholarships/degrees
  22.10	Has mechanisms to monitor research quality
  22.11	Has experts accessible for research advice
  22.12	Disseminates research results at research forums/seminars
  22.13	Supports an interdisciplinary approach to research
  22.14	Has incentives and support for mentoring activities
  22.15	Has external partners (e.g., government agencies, communities, public) engaged in research activities/planning
  22.16	Supports the peer-reviewed publication of research
  22.17	Has software available to support research activities
  22.18	Has adequate ethics support and planning
  22.19	Has adequate health and safety support and planning
  22.20	Has adequate data management support and planning
  22.21	Has adequate finance management support and planning

**23. What are the biggest barriers to research in your team?** Free text

**24. What are the biggest motivators to research in your team?** Free text

#### Section 4: Organisation level

**25.**

- **For each aspect, firstly rate *your perception* of your organisation’s (e.g., your University, Research Centre, NGO, etc.) success or skill level** (1 = no success/skill and 9 = highest possible success/skill): 1–9/unsure
- **And secondly, say whether you think this aspect has improved as a result of involvement with the Blue Communities project** (on a scale of much worse – worse – no change – better – much better/unsure)
  25.1	Has adequate resource to support staff research training
  25.2	Has funds, equipment or admin to support research activities
  25.3	Has a plan or policy for research development
  25.4	Has senior managers that support research
  25.5	Ensures staff career pathways are available in research
  25.6	Ensures organisational planning is guided by evidence
  25.7	Access external funding for research
  25.8	Encourages research activities relevant to creating impact (e.g., making a difference to society, SDGs, local communities, policies, management, etc.)
  25.9	Has software programs for analysing research data
  25.10	Has mechanisms to monitor research quality
  25.11	Has experts accessible for research advice
  25.12	Supports interdisciplinary approaches to research
  25.13	Has regular forums/bulletins to present research findings
  25.14	Engages external partners (e.g., government agencies, communities, public) in research activities/planning
  25.15	Supports applications for research scholarship/degrees
  25.16	Supports the peer-reviewed publication of research
  25.17	Has adequate ethics support and planning
  25.18	Has adequate health and safety support and planning
  25.19	Has adequate data management support and planning
  25.20	Has adequate finance management support and planning

**26. Any other comments:** Free text

### Appendix B: Tables

**Table A1.** Significant associations are highlighted between demographic variables based on Fisher’s exact test

Demographic variable	Category	Fisher’s exact test *p* value	Note
Gender	Age	0.009	More younger people are female
	Experience/Career stage	0.581	
	Contract	0.749	
	Country	0.083	
	Region	0.070	
Age	Experience/Career stage	0.004	Older people have more experience
	Contract	0.142	
	Country	0.432	
	Region	0.429	
Experience	Contract	0.063	
	Country	0.008	People with less experience more likely to be from Asia but experienced people from both
	Region	0.017	People with less experience more likely to be from Asia but experienced people from both
Contract	Country	0.317	
	Region	0.517	

**Table A2.** Significant associations are highlighted between individual level questions (linked to Figs 1–5 in the main text) with demographic variables based on Fisher’s exact test

Question	Demographic variable	Fisher’s exact test *p* value
Research Activity (Fig. 1)	Gender (removed ‘prefer not to say’)	0.987
	Age (removed ‘prefer not to say’)	0.984
	Experience [Very small categories combined, i.e., Undergraduate + Current MSc student; Post MSc (no PhD) + PhD student]	1.000
	Contract type	0.998
	Country	1.000
	Region	0.811
Resources (Fig. 2)	Gender (removed ‘prefer not to say’).	0.950
	Age (removed ‘prefer not to say’)	0.973
	Experience [Very small categories combined i.e., Undergraduate + Current MSc student; Post MSc (no PhD) + PhD student]	1.000
	Contract type	0.985
	Country	0.981
	Region	0.002
Research skills and opportunities valued (Fig. 3)	Gender (removed ‘prefer not to say’)	0.116
	Age (removed ‘prefer not to say’)	0.023
	Experience [Very small categories combined i.e., Undergraduate + Current MSc student; Post MSc (no PhD) + PhD student]	0.276
	Contract type	0.089
	Country	0.030
	Region	0.005
Barriers to research (Fig. 4)	Gender (removed ‘prefer not to say’)	0.365
	Age (removed ‘prefer not to say’)	0.131
	Experience [Very small categories combined i.e., Undergraduate + Current MSc student; Post MSc (no PhD) + PhD student]	0.949
	Contract type	0.009
	Country	0.015
	Region	0.001
Motivators (Fig. 5)	Gender (removed ‘prefer not to say’)	0.932
	Age (removed ‘prefer not to say’)	0.639
	Experience [Very small categories combined i.e., Undergraduate + Current MSc student; Post MSc (no PhD) + PhD student]	0.946
	Contract type	0.552
	Country	0.943
	Region	0.340

**Table A3.** Significant associations are highlighted between individual level questions (linked to Fig. 6 in the main text) with demographic variables based on Fisher’s exact test

Demographic variable	Letter code	Statement	Fisher’s exact test *p* value	Notes
Age	A	Relevant for impact	0.297	
	B	Rewarded by institution	0.472	
	C	Career progressed	0.192	
	D	Wrote grants	0.812	
	E	Interdisciplinary teams	0.011	Almost everyone agreed with this, older researchers agreed more strongly
	F	Carry on relationships	0.051	
	G	Lacking interdisciplinarity	0.358	
	H	Future career opportunities	0.052	
	I	Project managed	0.047	Those in older age categories agreed with this while others showed a range of responses
	J	Technical skills	0.113	
	K	Lead a publication	0.013	The youngest age category disagreed with this statement, while most others agreed
	L	My own research questions	0.105	
	M	Lead research	0.209	
	N	Training missing	0.100	
	O	Positive interdisciplinary working	0.044	Most strongly agreed with this, one group who preferred not to say their age were neutral/unsure
	P	Lack of time	0.274	
	Q	Could have led more	0.094	
Career/Experience	A	Relevant for impact	0.212	
	B	Rewarded by institution	0.295	
	C	Career progressed	0.397	
	D	Wrote grants	0.836	
	E	Interdisciplinary teams	0.559	
	F	Carry on relationships	0.894	
	G	Lacking interdisciplinarity	0.136	
	H	Future career opportunities	0.848	
	I	Project managed	0.259	
	J	Technical skills	0.196	
	K	Lead a publication	0.021	Most individuals from all career stage groups agreed with this, but individuals from the most experienced group and from the least experienced groups disagreed
	L	My own research questions	0.115	
	M	Lead research	0.828	
	N	Training missing	0.668	
	O	Positive interdisciplinary working	0.270	
	P	Lack of time	0.803	
	Q	Could have led more	0.048	PhD students and the most experienced researchers agreed that they could have led more
Contract	A	Relevant for impact	0.238	
	B	Rewarded by institution	0.103	
	C	Career progressed	0.847	
	D	Wrote grants	0.932	
	E	Interdisciplinary teams	0.671	
	F	Carry on relationships	0.438	
	G	Lacking interdisciplinarity	0.221	
	H	Future career opportunities	0.476	
	I	Project managed	0.362	
	J	Technical skills	0.440	
	K	Lead a publication	0.692	
	L	My own research questions	0.508	
	M	Lead research	0.236	
	N	Training missing	0.100	
	O	Positive interdisciplinary working	1.000	
	P	Lack of time	0.799	
	Q	Could have led more	0.477	
Gender	A	Relevant for impact	0.076	
	B	Rewarded by institution	0.369	
	C	Career progressed	0.227	
	D	Wrote grants	0.033	More males were neutral on this aspect, while females wither strongly disagreed or agreed and strongly agreed
	E	Interdisciplinary teams	0.045	More males strongly agree with this, while females mostly agreed or strongly agreed
	F	Carry on relationships	0.463	
	G	Lacking interdisciplinarity	0.449	
	H	Future career opportunities	0.038	More males strongly agree with this, while females mostly agreed or strongly agreed
	I	Project managed	0.789	
	J	Technical skills	0.178	
	K	Lead a publication	0.602	
	L	My own research questions	0.152	
	M	Lead research	0.957	
	N	Training missing	0.491	
	O	Positive interdisciplinary working	0.005	More males strongly agree with this, while females mostly agreed or strongly agreed
	P	Lack of time	0.456	
	Q	Could have led more	0.104	
Region	A	Relevant for impact	0.001	SE Asia researchers mostly strongly agreed, more UK researchers gave a neutral response	
	B	Rewarded by institution	0.818	
	C	Career progressed	0.041	SE Asia researchers mostly strongly agreed, more UK researchers gave a neutral response
	D	Wrote grants	0.104	
	E	Interdisciplinary teams	1.000	
	F	Carry on relationships	0.374	
	G	Lacking interdisciplinarity	0.206	
	H	Future career opportunities	0.016	SE Asia researchers mostly strongly agreed, more UK researchers gave a neutral response
	I	Project managed	0.535	
	J	Technical skills	0.001	SE Asia researchers mostly strongly agreed, more UK researchers gave a neutral response
	K	Lead a publication	0.113	
	L	My own research questions	0.009	SE Asia researchers mostly strongly agreed, more UK researchers gave a neutral response
	M	Lead research	0.600	
	N	Training missing	0.665	
	O	Positive interdisciplinary working	0.512	
	P	Lack of time	0.603	
	Q	Could have led more	0.043	SE Asia researchers mostly responded neutrally, while UK researchers gave a range of responses here, but none strongly agreed

**Table A4.** Codes, full statement and percentage level of agreement associated with Fig. 6 in the main text

Letter code given in Fig. 6	Full statement associated with code	Group	Level of agreement as percentage of group					
			Strongly disagree	Disagree	Neutral	Agree	Strongly agree	Don’t know
A	The research I carried out during Blue Communities was relevant to creating impact (e.g., making a difference to society, SDGs, local communities, policies, management, etc.) in my region	Full Group	3.6	0.0	12.5	35.7	48.2	0.0
		UK/Other European	5.6	0.0	38.9	44.4	11.1	0.0
		SE Asia	2.6	0.0	0.0	31.6	65.8	0.0
B	My institution rewards or recognises my achievements linked to BC	Full Group	7.1	1.8	25.0	32.1	26.8	7.1
		UK/Other European	5.6	5.6	27.8	27.8	33.3	0.0
		SE Asia	7.9	0.0	23.7	34.2	23.7	10.5
C	My career level has progressed as a result of my involvement in BC	Full Group	1.8	1.8	19.6	32.1	44.6	0.0
		UK/Other European	0.0	5.6	38.9	27.8	27.8	0.0
		SE Asia	2.6	0.0	10.5	34.2	52.6	0.0
D	I wrote new research grants during my time on BC	Full Group	7.1	14.3	23.2	19.6	33.9	1.8
		UK/Other European	16.7	16.7	5.6	33.3	27.8	0.0
		SE Asia	2.6	13.2	31.6	13.2	36.8	2.6
E	I worked with interdisciplinary teams	Full Group	1.8	0.0	5.4	25.0	66.1	1.8
		UK/Other European	0.0	0.0	11.1	22.2	61.1	5.6
		SE Asia	2.6	0.0	2.6	26.3	68.4	0.0
F	I will build upon the international networks and professional relationships that have been developed through the BC programme	Full Group	0.0	1.8	17.9	26.8	50.0	3.6
		UK/Other European	0.0	0.0	16.7	38.9	33.3	11.1
		SE Asia	0.0	2.6	18.4	21.1	57.9	0.0
G	I thought the BC research could have been more interdisciplinary	Full Group	7.1	28.6	23.2	19.6	17.9	3.6
		UK/Other European	5.6	50.0	0.0	11.1	22.2	11.1
		SE Asia	7.9	18.4	34.2	23.7	15.8	0.0
H	I think I will have more opportunities available to enhance my future career as a result of the work I have conducted for the BC programme	Full Group	1.8	1.8	10.7	33.9	51.8	0.0
		UK/Other European	0.0	5.6	27.8	33.3	33.3	0.0
		SE Asia	2.6	0.0	2.6	34.2	60.5	0.0
I	I project-managed	Full Group	7.1	1.8	14.3	39.3	37.5	0.0
		UK/Other European	11.1	0.0	5.6	50.0	33.3	0.0
		SE Asia	5.3	2.6	18.4	34.2	39.5	0.0
J	I learned new technical specialist skills	Full Group	1.8	1.8	12.5	37.5	46.4	0.0
		UK/Other European	0.0	5.6	33.3	55.6	5.6	0.0
		SE Asia	2.6	0.0	2.6	28.9	65.8	0.0
K	I have had the opportunity to be the lead author on one/more than one publication	Full Group	7.1	7.1	17.9	19.6	48.2	0.0
		UK/Other European	5.6	16.7	27.8	22.2	27.8	0.0
		SE Asia	7.9	2.6	13.2	18.4	57.9	0.0
L	I have been able to answer some of my own research questions	Full Group	0.0	3.6	16.1	39.3	41.1	0.0
		UK/Other European	0.0	5.6	38.9	27.8	27.8	0.0
		SE Asia	0.0	2.6	5.3	44.7	47.4	0.0
M	I had the opportunity to lead research work and/or contribute ideas that directed the research	Full Group	3.6	1.8	5.4	28.6	60.7	0.0
		UK/Other European	0.0	0.0	11.1	33.3	55.6	0.0
		SE Asia	5.3	2.6	2.6	26.3	63.2	0.0
N	I felt some types of training were missing from the BC project	Full Group	5.4	26.8	37.5	19.6	5.4	5.4
		UK/Other European	0.0	50.0	22.2	16.7	5.6	5.6
		SE Asia	7.9	15.8	44.7	21.1	5.3	5.3
O	I feel positive about working with people from different disciplines in the future	Full Group	0.0	1.8	5.4	17.9	73.2	1.8
		UK/Other European	0.0	0.0	11.1	11.1	72.2	5.6
		SE Asia	0.0	2.6	2.6	21.1	73.7	0.0
P	I did not have time to learn all that I might have during BC	Full Group	5.4	12.5	17.9	42.9	21.4	0.0
		UK/Other European	0.0	5.6	16.7	55.6	22.2	0.0
		SE Asia	7.9	15.8	18.4	36.8	21.1	0.0
Q	I could have led more work than I did during the BC project	Full Group	14.3	17.9	30.4	30.4	7.1	0.0
		UK/Other European	16.7	33.3	11.1	38.9	0.0	0.0
		SE Asia	13.2	10.5	39.5	26.3	10.5	0.0

**Table A5.** Significant associations are highlighted between individual level questions (linked to Fig. 7 in the main text) with demographic variables based on Fisher’s exact test

Demographic variable	Letter code	Aspect of research capacity	Success level Fisher’s exact test *p* value	Improvement level Fisher’s exact test *p* value	Explanatory notes
Age	A	Qualitative analysis	0.378	0.497	
	B	Quantitative analysis	0.150	0.900	
	C	Apply funding	0.386	0.578	
	D	Data collection	0.048	0.178	31–50-year-olds scored better overall
	E	Review literature	0.036	0.789	Older age categories scored better
	F	Questionnaires	0.360	0.573	
	G	Finding literature	0.062	0.185	
	H	Manage a project	0.283	0.597	
	I	Networking	0.816	0.538	
	J	Present research	0.408	0.139	
	K	Provide advice	0.204	0.253	
	L	Secure grants	0.789	0.217	
	M	Health and safety	0.854	0.638	
	N	Ethics	0.470	0.292	
	O	Finance claims	0.795	0.378	
	P	Interdisciplinary approaches	0.669	0.585	
	Q	Overseas issues	0.589	0.438	
	R	Referencing system	0.552	0.852	
	S	Data management	0.114	0.571	
	T	Protocol or study design	0.600	0.664	
	U	Research report	0.226	0.490	
	V	Publication	0.344	0.502	
Career	A	Qualitative analysis	0.555	0.827	
	B	Quantitative analysis	0.228	0.409	
	C	Apply funding	0.418	0.737	
	D	Data collection	0.439	0.269	
	E	Review literature	0.108	0.176	
	F	Questionnaires	0.502	0.895	
	G	Finding literature	0.015	0.056	More early career researchers (up to PhD student) scored themselves lower on this
	H	Manage a project	0.263	0.997	
	I	Networking	0.928	0.191	
	J	Present research	0.813	0.961	
	K	Provide advice	0.175	0.413	
	L	Secure grants	0.077	0.141	
	M	Health and safety	0.201	0.409	
	N	Ethics	0.695	0.295	
	O	Finance claims	0.283	0.994	
	P	Interdisciplinary approach…	0.535	0.872	
	Q	Overseas issues	0.257	0.398	
	R	Referencing system	0.165	0.058	
	S	Data management	0.266	0.937	
	T	Protocol or study design	0.866	0.965	
	U	Research report	0.172	0.407	
	V	Publication	0.037	0.640	More early career researchers (up to PhD student) scored themselves lower on this
Contract	A	Qualitative analysis	0.894	0.732	
	B	Quantitative analysis	0.961	0.298	
	C	Apply funding	0.365	0.295	
	D	Data collection	0.954	0.148	
	E	Review literature	0.360	1.000	
	F	Questionnaires	0.819	0.582	
	G	Finding literature	0.076	0.557	
	H	Manage a project	0.320	1.000	
	I	Networking	0.143	0.370	
	J	Present research	0.402	0.363	
	K	Provide advice	0.717	1.000	
	L	Secure grants	0.752	0.334	
	M	Health and safety	0.193	0.356	
	N	Ethics	0.871	0.295	
	O	Finance claims	0.199	0.405	
	P	Interdisciplinary approaches	0.193	0.420	
	Q	Overseas issues	0.344	1.000	
	R	Referencing system	0.848	0.106	
	S	Data management	0.622	0.411	
	T	Protocol or study design	0.957	0.536	
	U	Research report	0.589	0.649	
	V	Publication	0.899	0.822	
Gender	A	Qualitative analysis	0.226	0.289	
	B	Quantitative analysis	0.001	0.135	Most males and females scored themselves mid-high on this, but some females scored themselves very low on this
	C	Apply funding	0.408	0.598	
	D	Data collection	0.294	0.282	
	E	Review literature	0.523	0.110	
	F	Questionnaires	0.328	0.215	
	G	Finding literature	0.850	0.214	
	H	Manage a project	0.552	0.957	
	I	Networking	0.731	0.233	
	J	Present research	0.589	0.654	
	K	Provide advice	0.757	0.431	
	L	Secure grants	0.896	0.339	
	M	Health and safety	0.338	0.509	
	N	Ethics	0.824	0.768	
	O	Finance claims	0.868	0.135	
	P	Interdisciplinary approaches	0.854	0.110	
	Q	Overseas issues	0.092	0.359	
	R	Referencing system	0.217	0.718	
	S	Data management	0.416	0.221	
	T	Protocol or study design	0.755	0.240	
	U	Research report	0.864	0.485	
	V	Publication	0.153	0.633	
Region	A	Qualitative analysis	0.523	0.021	SE Asia researchers indicated higher improvement, while UK researchers indicated no change or lower degree of improvement
	B	Quantitative analysis	0.351	0.028	
	C	Apply funding	0.371	0.229	
	D	Data collection	0.074	0.001	SE Asia researchers indicated higher improvement, while UK researchers indicated no change or lower degree of improvement
	E	Review literature	0.688	0.001	
	F	Questionnaires	0.560	0.001	
	G	Finding literature	0.870	0.001	
	H	Manage a project	0.085	0.018	
	I	Networking	0.244	0.001	
	J	Present research	0.446	0.008	
	K	Provide advice	0.955	0.380	
	L	Secure grants	0.605	0.301	
	M	Health and safety	0.090	0.514	
	N	Ethics	0.899	0.124	
	O	Finance claims	0.356	0.135	
	P	Interdisciplinary approaches	0.531	0.001	SE Asia researchers indicated higher improvement, while UK researchers indicated no change or lower degree of improvement
	Q	Overseas issues	0.444	0.848	
	R	Referencing system	0.287	0.001	SE Asia researchers indicated higher improvement, while UK researchers indicated no change or lower degree of improvement
	S	Data management	0.687	0.027	
	T	Protocol or study design	0.525	0.083	
	U	Research report	0.887	0.002	SE Asia researchers indicated higher improvement, while UK researchers indicated no change or lower degree of improvement
	V	Publication	0.818	0.008	

**Table A6.** Codes and full description of aspect of research capacity associated with Fig. 7 in the main text

Letter code given in Fig. 7	Full research capacity aspect associated with code
A	Analysing qualitative research data
B	Analysing quantitative research data
C	Applying for research funding/writing research grants
D	Collecting data, e.g., surveys, interviews
E	Critically reviewing the literature
F	Designing questionnaires
G	Finding relevant literature
H	Managing a project
I	Networking
J	Presenting research findings
K	Providing advice to less experienced researchers
L	Securing research funding
M	Submitting a health and safety assessment
N	Submitting an ethics application
O	Submitting financial claims from a research grant
P	Understanding interdisciplinary approaches and issues
Q	Understanding overseas issues and challenges
R	Using a computer referencing system (e.g., Endnote)
S	Using computer data management systems
T	Writing a research protocol or designing a study
U	Writing a research report
V	Writing for publication in peer-reviewed journals

**Table A7.** Significant associations are highlighted between team level questions (linked to Fig. 8 in the main text) with demographic variables based on Fisher’s exact test

Demographic variable	Letter code	Aspect of research capacity	Success level Fisher’s exact test *p* value	Improvement level Fisher’s exact test *p* value	Notes
Age	A	Impactful research	0.978	0.886	
	B	Disseminates research	0.997	0.658	
	C	Planning with evidence	0.993	0.619	
	D	Team level planning	0.958	0.817	
	E	Staff involved in plans	0.990	0.820	
	F	Data management	0.921	0.500	
	G	Ethics	0.664	0.445	
	H	Finance management	0.894	0.356	
	I	Health and safety	0.942	0.191	
	J	Staff training	0.183	0.867	
	K	Expert advice	0.913	0.896	
	L	External partners	0.911	0.922	
	M	Funds, equipment, admin	0.831	0.541	
	N	Mentoring	0.706	0.945	
	O	Research quality	0.986	0.359	
	P	Software	0.974	0.138	
	Q	Leaders support research	0.931	0.799	
	R	Research opportunities	0.950	0.360	
	S	Interdisciplinary approach	0.957	0.503	
	T	Scholarships	0.100	0.872	
	U	Publication	0.339	0.450	
Career	A	Impactful research	0.733	0.995	
	B	Disseminates research	0.044	0.978	Early career, students and <5 years post PhD scored their teams highly on this
	C	Planning with evidence	0.418	0.276	
	D	Team level planning	0.586	0.753	
	E	Staff involved in plans	0.700	0.826	
	F	Data management	0.696	0.838	
	G	Ethics	0.104	0.214	
	H	Finance management	0.305	0.695	
	I	Health and safety	0.623	0.333	
	J	Staff training	0.818	0.888	
	K	Expert advice	0.010	0.530	PhD students scored their teams lower on this
	L	External partners	0.722	0.648	
	M	Funds, equipment, admin	0.431	0.880	
	N	Mentoring	0.283	0.420	
	O	Research quality	0.128	0.821	
	P	Software	0.007	0.352	More experienced researchers scored their teams higher on this than early and mid-career researchers
	Q	Leaders support research	0.346	0.747	
	R	Research opportunities	0.054	0.808	
	S	Interdisciplinary approach	0.293	0.876	
	T	Scholarships	0.041	0.665	Some early career groups – PhD students and up to 5 years post PhD – scored their teams lower on this than other groups
	U	Publication	0.388	0.180	
Contract	A	Impactful research	0.386	0.798	
	B	Disseminates research	0.187	0.551	
	C	Planning with evidence	0.647	0.766	
	D	Team level planning	0.592	0.798	
	E	Staff involved in plans	0.494	0.699	
	F	Data management	0.063	0.940	
	G	Ethics	0.946	0.420	
	H	Finance management	0.801	0.724	
	I	Health and safety	0.544	0.191	
	J	Staff training	0.886	0.564	
	K	Expert advice	0.873	0.683	
	L	External partners	0.980	1.000	
	M	Funds, equipment, admin	0.539	0.930	
	N	Mentoring	0.107	0.100	
	O	Research quality	0.703	0.933	
	P	Software	0.035	0.619	Some of those on fixed-term contracts scored their teams lower than those on permanent contracts
	Q	Leaders support research	0.567	0.929	
	R	Research opportunities	0.733	0.487	
	S	Interdisciplinary approach	0.129	0.742	
	T	Scholarships	0.920	1.000	
	U	Publication	0.522	0.938	
Gender	A	Impactful research	0.905	0.588	
	B	Disseminates research	0.715	0.549	
	C	Planning with evidence	0.622	0.358	
	D	Team level planning	0.685	0.403	
	E	Staff involved in plans	0.547	0.606	
	F	Data management	0.448	0.684	
	G	Ethics	0.101	0.209	
	H	Finance management	0.279	0.271	
	I	Health and safety	0.078	0.870	
	J	Staff training	0.902	0.711	
	K	Expert advice	0.608	0.108	
	L	External partners	0.025	0.916	More male researchers scored their teams lower on this
	M	Funds, equipment, admin	0.458	0.518	
	N	Mentoring	0.284	0.354	
	O	Research quality	0.842	0.904	
	P	Software	0.171	0.720	
	Q	Leaders support research	0.465	0.839	
	R	Research opportunities	0.917	0.554	
	S	Interdisciplinary approach	0.686	0.267	
	T	Scholarships	0.297	0.188	
	U	Publication	0.074	0.588	
Region	A	Impactful research	0.519	0.024	SE Asia researchers indicated higher improvement, while UK researchers indicated no change or lower degree of improvement
	B	Disseminates research	0.199	0.001	
	C	Planning with evidence	0.932	0.003	
	D	Team level planning	0.663	0.001	
	E	Staff involved in plans	0.102	0.001	
	F	Data management	0.840	0.001	
	G	Ethics	0.710	0.001	
	H	Finance management	0.629	0.001	
	I	Health and safety	0.651	0.001	
	J	Staff training	0.375	0.003	
	K	Expert advice	0.527	0.001	
	L	External partners	0.100	0.001	
	M	Funds, equipment, admin	0.438	0.001	
	N	Mentoring	0.765	0.020	
	O	Research quality	0.817	0.009	
	P	Software	0.486	0.004	
	Q	Leaders support research	0.290	0.001	
	R	Research opportunities	0.261	0.001	
	S	Interdisciplinary approach	0.239	0.001	
	T	Scholarships	0.503	0.070	
	U	Publication	0.365	0.001	SE Asia researchers indicated higher improvement, while UK researchers indicated no change or lower degree of improvement

**Table A8.** Codes and full description of aspect of research capacity associated with Fig. 8 in the main text

Letter code given in Fig. 8	Full research capacity aspect associated with code
A	Conducts research activities relevant to creating impact (e.g., making a difference to society, SDGs, local communities, policies, management, etc.)
B	Disseminates research results at research forums/seminars
C	Does planning that is guided by evidence
D	Does team level planning for research development
E	Ensures staff involvement in developing that plan
F	Has adequate data management support and planning
G	Has adequate ethics support and planning
H	Has adequate finance management support and planning
I	Has adequate health and safety support and planning
J	Has adequate resources to support staff research training
K	Has experts accessible for research advice
L	Has external partners (e.g., government agencies, communities, public) engaged in research activities/planning
M	Has funds, equipment or admin to support research activities
N	Has incentives and support for mentoring activities
O	Has mechanisms to monitor research quality
P	Has software available to support research activities
Q	Has team leaders that support research
R	Provides opportunities to get involved in research
S	Supports an interdisciplinary approach to research
T	Supports applications for research scholarships/degrees
U	Supports the peer-reviewed publication of research

**Table A9.** Significant associations are highlighted between institution level questions (linked to Fig. 9 in the main text) with demographic variables based on Fisher’s exact test

Demographic variable	Letter code	Aspect of Research Capacity	Success level Fisher’s exact test *p* value	Improvement level Fisher’s exact test *p* value	Notes
Age	A	External funding	0.893	0.537	
	B	Impactful research	0.501	0.699	
	C	External partners	0.188	0.112	
	D	Planning with evidence	0.139	0.950	
	E	Career pathways	0.382	0.683	
	F	Research development policy	0.861	0.582	
	G	Data management	0.565	0.212	
	H	Ethics	0.667	0.979	
	I	Finance management	0.863	0.290	
	J	Health and safety	0.396	0.962	
	K	Staff training	0.990	0.976	
	L	Experts	0.960	0.322	
	M	Funds, equipment, admin	0.911	0.728	
	N	Research quality	0.698	0.270	
	O	Dissemination	0.755	0.898	
	P	Leaders support research	0.335	0.825	
	Q	Software	0.642	0.386	
	R	Scholarships	0.627	0.954	
	S	Interdisciplinary approach	0.584	0.713	
	T	Publication	0.453	0.612	
Career	A	External funding	0.046	0.485	Early–mid (post MSc up to 15 years post PhD) level were more likely to score their institution lower on this
	B	Impactful research	0.853	0.455	
	C	External partners	0.074	0.194	
	D	Planning with evidence	0.285	0.372	
	E	Career pathways	0.179	0.453	
	F	Research development policy	0.578	0.938	
	G	Data management	0.551	0.855	
	H	Ethics	0.088	0.498	
	I	Finance management	0.214	0.433	
	J	Health and safety	0.186	0.236	
	K	Staff training	0.199	0.366	
	L	Experts	0.255	0.278	
	M	Funds, equipment, admin	0.693	0.451	
	N	Research quality	0.280	0.722	
	O	Dissemination	0.116	0.533	
	P	Leaders support research	0.702	0.298	
	Q	Software	0.011	0.090	Later career (more than 15 years post PhD) were more likely to score their institution higher on this
	R	Scholarships	0.236	0.428	
	S	Interdisciplinary approach	0.042	0.772	Later career (more than 15 years post PhD) were more likely to score their institution higher on this
	T	Publication	0.198	0.688	
Contract	A	External funding	0.672	0.626	
	B	Impactful research	0.807	0.700	
	C	External partners	0.964	0.969	
	D	Planning with evidence	0.185	0.834	
	E	Career pathways	0.233	0.417	
	F	Research development policy	0.300	0.681	
	G	Data management	0.749	0.717	
	H	Ethics	0.864	0.770	
	I	Finance management	0.923	0.717	
	J	Health and safety	0.986	0.435	
	K	Staff training	0.701	1.000	
	L	Experts	0.372	0.897	
	M	Funds, equipment, admin	0.387	0.929	
	N	Research quality	0.838	0.294	
	O	Dissemination	0.541	0.936	
	P	Leaders support research	0.847	0.676	
	Q	Software	0.140	0.237	
	R	Scholarships	0.908	0.454	
	S	Interdisciplinary approach	0.933	0.628	
	T	Publication	0.290	1.000	
Gender	A	External funding	0.630	0.683	
	B	Impactful research	0.298	0.100	
	C	External partners	0.650	0.313	
	D	Planning with evidence	0.449	0.154	
	E	Career pathways	0.553	0.087	
	F	Research development policy	0.765	0.007	Females were more likely to report no improvement on this aspect in their institution
	G	Data management	0.446	0.115	
	H	Ethics	0.981	0.006	Females were more likely to report no improvement on this aspect in their institution
	I	Finance management	0.597	0.408	
	J	Health and safety	0.780	0.558	
	K	Staff training	0.976	0.229	
	L	Experts	0.796	0.407	
	M	Funds, equipment, admin	0.822	0.393	
	N	Research quality	0.928	0.479	
	O	Dissemination	0.974	0.854	
	P	Leaders support research	0.971	0.420	
	Q	Software	0.624	0.796	
	R	Scholarships	0.999	0.329	
	S	Interdisciplinary approach	0.590	0.595	
	T	Publication	0.503	0.639	
Region	A	External funding	0.931	0.001	SE Asia researchers indicated higher improvement, while UK researchers indicated no change or lower degree of improvement
	B	Impactful research	0.879	0.003	
	C	External partners	0.905	0.002	
	D	Planning with evidence	0.960	0.001	
	E	Career pathways	0.762	0.001	
	F	Research development policy	0.932	0.001	
	G	Data management	0.988	0.001	
	H	Ethics	0.501	0.001	
	I	Finance management	0.972	0.001	
	J	Health and safety	0.695	0.001	
	K	Staff training	0.050	0.001	UK researchers were more likely to score a high score (above 7) for their institutions on this. Several SE Asian researchers scored their institutions mid (5–7) on this, though some also scored gave the highest score. SE Asia researchers indicated higher improvement, while UK researchers indicated no change or lower degree of improvement
	L	Experts	0.952	0.002	SE Asia researchers indicated higher improvement, while UK researchers indicated no change or lower degree of improvement
	M	Funds, equipment, admin	0.313	0.001	
	N	Research quality	1.000	0.001	
	O	Dissemination	0.886	0.008	
	P	Leaders support research	0.384	0.001	
	Q	Software	0.806	0.013	
	R	Scholarships	1.000	0.001	
	S	Interdisciplinary approach	0.744	0.002	
	T	Publication	0.888	0.001	

**Table A10.** Codes and full description of aspect of research capacity associated with Fig. 9 in the main text

Letter code given in Fig. 9	Full research capacity aspect associated with code
A	Access external funding for research
B	Encourages research activities relevant to creating impact (e.g., making a difference to society, SDGs, local communities, policies, management, etc.)
C	Engages external partners (e.g., government agencies, communities, public) in research activities/planning
D	Ensures organisational planning is guided by evidence
E	Ensures staff career pathways are available in research
F	Has a plan or policy for research development
G	Has adequate data management support and planning
H	Has adequate ethics support and planning
I	Has adequate finance management support and planning
J	Has adequate health and safety support and planning
K	Has adequate resource to support staff research training
L	Has experts accessible for research advice
M	Has funds, equipment or admin to support research activities
N	Has mechanisms to monitor research quality
O	Has regular forums/bulletins to present research findings
P	Has senior managers that support research
Q	Has software programs for analysing research data
R	Supports applications for research scholarship/degrees
S	Supports interdisciplinary approaches to research
T	Supports the peer-reviewed publication of research
